# International Standards for Neurological Classification of Spinal Cord Injury: Case Examples Reinforcing Concepts From the 2019 Revision

**DOI:** 10.46292/sci24-00049

**Published:** 2025-08-22

**Authors:** Brittany Snider, Steven Kirshblum, Ruediger Rupp, Christian Schuld, Fin Biering-Sorensen, Stephen Burns, James Guest, Linda Jones, Andrei Krassioukov, Gianna Rodriguez, Mary Schmidt Read, Keith Tansey, Kristen Walden

**Affiliations:** 1 Kessler Institute for Rehabilitation, West Orange, New Jersey; 2 Rutgers New Jersey Medical School, Department of Physical Medicine & Rehabilitation, Newark, New Jersey; 3 Spinal Cord Injury Center, Heidelberg University Hospital, Heidelberg, Germany; 4 Medical Faculty Heidelberg, Heidelberg University, Heidelberg, Germany; 5 Department of Clinical Medicine, University of Copenhagen, and Department of Brain and Spinal Cord Injuries, Rigshospitalet, Copenhagen, Denmark; 6 Department of Rehabilitation Medicine, University of Washington School of Medicine, Seattle, Washington; 7 University of Miami, Miller School of Medicine, Department of Neurological Surgery, Miami, Florida; 8 Thomas Jefferson University, Philadelphia, Philadelphia; 9 International Collaboration on Repair Discovery (ICORD), University of British Columbia, Vancouver, British Columbia, Canada; 10 Michigan Medicine, Department of Physical Medicine and Rehabilitation, Ann Arbor, Michigan; 11 Magee Rehabilitation Hospital, Jefferson Health, Philadelphia, Pennsylvania; 12 Center for Neuroscience and Neurological Recovery, Methodist Rehabilitation Center, Jackson, Mississippi; 13 Spinal Cord Injury Medicine and Research Services, Jackson Veterans Administration Medical Center, Jackson, Mississippi; 14 Department of Neurosurgery, University of Mississippi Medical Center, Jackson, Mississippi; 15 Praxis Spinal Cord Institute, Vancouver, Canada

**Keywords:** classification accuracy, International Standards for Neurological Classification of Spinal Cord Injury (ISNCSCI), spinal cord injury

## Abstract

**Background:**

The International Standards for Neurological Classification of Spinal Cord Injury (ISNCSCI) is the most widely accepted system for characterizing sensorimotor impairments after spinal cord injury (SCI). There have been a number of ISNCSCI revisions, with the most recent edition published in 2019. Newer concepts, including the revised definitions of the zones of partial preservation (ZPPs) and documentation of non-SCI conditions, require training and practice for successful utilization. The International Standards Committee developed an ISNCSCI workbook of 26 practice cases, each with detailed explanations of the correct classification components. In this article, we present seven cases, which were selected from the workbook to reinforce the changes implemented in 2019.

**Methods:**

Hypothetical ISNCSCI cases were created to illustrate important classification rules, definitions, and nuances. All cases were reviewed by members of the American Spinal Injury Association (ASIA) International Standards Committee, and if any discrepancies were identified, they were discussed until a consensus was reached. To confirm agreement, cases were also entered into online algorithms, which are compliant with the 2019 ISNCSCI revision. The seven cases in this article highlight newer classification concepts and include a discussion of key elements.

**Cases:**

Each case reinforces the revised definitions of the ZPPs, such as the applicability of sensory ZPPs in all injuries without sensory sacral sparing and applicability of motor ZPPs in all injuries without voluntary anal contraction (VAC). Non-SCI-related impairments and their impact on the classification are reviewed in Cases 4-7.

**Conclusion:**

The seven cases presented in this article feature key concepts from the 2019 ISNCSCI revision. These cases, as well as the full ISNCSCI workbook, can serve as valuable training tools to improve classification accuracy.

## Introduction

The International Standards for Neurological Classification of Spinal Cord Injury (ISNCSCI) is the most widely accepted taxonomy for characterizing sensorimotor impairments after spinal cord injury (SCI). Since its inception in 1982,^1^ there have been a number of ISNCSCI revisions, with the newest edition published in 2019.^2^ The ISNCSCI is maintained by the International Standards Committee of the American Spinal Injury Association (ASIA). Accurate classification is essential as the ISNCSCI is used to monitor changes in sensory and motor function over time, establish realistic rehabilitation goals and effective therapy programs, determine clinical trial eligibility, and predict the probability of neurological recovery.

SCI is a heterogeneous condition, and even within the same ASIA Impairment Scale (AIS) grade, there are variations in clinical presentation and outcomes. The ISNCSCI has been refined throughout the years to more accurately characterize each unique spinal cord lesion. As such, ISNCSCI classification involves precise rules and nuances, and inherent challenges have been described.^3^-^5^ Compared to neurological complete (AIS A) injuries, incomplete lesions tend to be more difficult to classify,^6^,^7^ and classification components associated with the highest error rates include motor levels, AIS grade, and zones of partial preservation (ZPPs).^6^,^8^-^11^ A recent study evaluating classification accuracy of SCI professionals (most of whom rated themselves as experienced examiners) found that newer concepts introduced with the 2019 revision, including the revised definitions of the ZPPs and documentation of non-SCI conditions, contributed to several common mistakes.^11^

Below is a summary of the two major changes introduced in the 2019 revision.

1. *ZPPs:* In injuries without sensory and/or motor sacral sparing, the sensory and/or motor ZPPs represent the most caudal dermatome and/or myotome on each side with partially preserved functions. Prior to 2019, the ZPPs were only applicable in complete (AIS A) injuries, which have absent sensory *and* motor functions in the lowest sacral segments. The new ZPP rule states that a sensory ZPP is applicable in all injuries without sensory function in the lowest sacral segments. A sensory ZPP is not applicable (NA) if there is preservation of deep anal pressure (DAP), pin prick (PP) sensation, or light touch (LT) sensation in the S4-5 dermatome. The sensory ZPPs are determined independently for the right and left sides, and it is possible for the sensory ZPP to be NA on one side and applicable on the other (e.g., in an injury with absent DAP sensation but with preservation of S4-5 PP/LT sensation on only one side). Additionally, motor ZPPs are determined separately for each side and are applicable bilaterally in all injuries with absent voluntary anal contraction (VAC).^2^,^12^,^13^
2. *Non-SCI conditions:* If sensorimotor functions are thought to be impacted by a non-SCI condition (i.e., pain, fracture, burn, peripheral nerve injury, etc.), the examiner should record the actual examined scores and tag them with an “*”. The non-SCI condition is then documented in the comments box with the recommendation for how the “*”-tagged scores should be treated during classification. This includes whether they are considered normal (typically rostral to the neurological level of injury [NLI]) or not normal (in most cases at or caudal to the NLI) for classification. Note that only impaired sensory and motor scores are “*”-tagged (there is no 2* for the sensory exam or 5* for the motor exam). The presence of “*”-tagged scores may influence the classification of levels, completeness, AIS grade, and ZPPs. If the classification of any of these variables is based on a clinical assumption, the classification component should be tagged with an “*”. More detailed explanations of the rules for the documentation of non-SCI-related impairments can be found in previous publications.^2^,^12^-^14^

Both concepts introduced in 2019 have clinical value and enhance the information obtained using the ISNCSCI. The ZPPs describe the extent of preserved neurological function and, along with the other classification variables, help to provide a comprehensive characterization of neurological impairment after SCI. The ZPPs also address the issue of within-group heterogeneity. For example, the motor ZPP would distinguish a C3 AIS A injury with motor preservation to the C7 segment (motor ZPP of C7) from a C3 AIS A injury with no motor preservation below the motor level (motor ZPP of C3). With the 2019 revision, this value has been extended to incomplete injuries with absent sensory and/or motor function in the lowest sacral segments, and the expanded definition is especially useful in describing the motor ZPP in AIS B injuries (and rare motor incomplete injuries) with absent VAC.^15^,^16^ The updated taxonomy for non-SCI conditions has a number of benefits as well. Previously, a motor grade of “5*” was assigned to key muscles when the examiner believed motor function would have been intact if not for a non-SCI condition. There was no system for documenting impairments thought to be due to both the SCI and non-SCI condition (e.g., peroneal neuropathy and incomplete tetraplegia [see Case 7]), and there was also no method for documenting non-SCI-related sensory impairments. The new taxonomy overcomes these limitations. By recording the actual examined scores, relevant clinical information is preserved, and serial assessments are possible. Additionally, application of the “*” concept to the classification system is useful as it draws attention to the non-SCI condition (if assumptions about the non-SCI condition impact the classifications) and provides a more accurate description of the SCI in such cases. Furthermore, in research settings, it may increase clinical trial eligibility for those with a non-SCI condition who would have otherwise been excluded, as there is now a more systematic way of handling non-SCI-related impairments.^14^

The International Standards Committee developed an ISNCSCI workbook^17^ of 26 practice cases with detailed explanations of the correct classifications. The workbook is available for download on the ASIA website^17^ and is also published in electronic form under an open access license in the Open Data Commons for Spinal Cord Injury (ODC-SCI).^18^ It can serve as a reference for calculator developers and ISNCSCI teachers as well as a training tool for persons interested in enhancing their ISNCSCI classification skills. In this article, we present seven cases that were selected from the workbook to reinforce the changes implemented in 2019. The primary goals of this work are increased utilization and understanding of the current ISNCSCI edition.

## Methods

Twenty-six hypothetical ISNCSCI cases were created by the authors to illustrate important classification rules, definitions, and nuances. Many of the cases were compiled to address specific questions received after ISNCSCI trainings, webinars, “Ask the Expert” calls, and so on. All cases were reviewed by members of the International Standards Committee. To confirm agreement, cases were also entered into online algorithms,^19^,^20^ which are compliant with the 2019 ISNCSCI revision. If any discrepancies were identified, they were discussed by members of the committee until a consensus was reached.

The seven representative cases in this article highlight newer classification concepts, such as revised definitions for the sensory and motor ZPPs and taxonomy of non-SCI impairments. The cases were selected by the authors to address a variety of teaching points and potentially challenging concepts. These include, but are not limited to, the classification of ZPPs in incomplete injuries, the role of non-key muscle function in determining motor incomplete status and motor ZPP, not testable* (NT*) examination components due to a non-SCI condition, and classification components that are not determinable (ND or ND*). Another consideration in case selection was the location of the non-SCI condition in relation to the NLI, as this often impacts the clinical assumptions and classification results. ISNCSCI worksheets for these cases are presented below. Each case includes a review of the correct classification components (right/left sensory levels, right/left motor levels, NLI, completeness, AIS grade, right/left sensory ZPPs, and right/left motor ZPPs) and discussion of key elements.

## Cases

### Case 1: Use of the ZPPs and classification of a motor incomplete injury in the absence of VAC.

**Figure:**
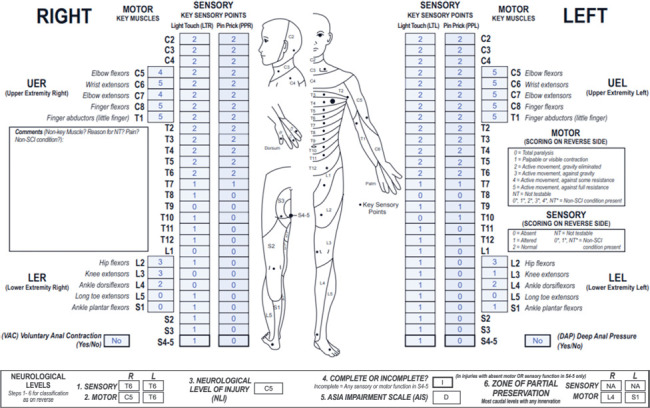
This case represents a cervical motor incomplete SCI with sensory ZPPs that are NA due to preserved sensory sacral sparing bilaterally and motor ZPPs that are applicable as VAC is absent.

#### Sensory levels:

The sensory level is T6 bilaterally as sensory function is intact from C2 through this dermatome on both sides.

#### Motor levels:

The right motor level is C5 because this is the most caudal key muscle with a grade ≥3, and motor function above this level is presumed to be intact. Although there is no testable motor function at the C2-C4 myotomes, the motor grade at each of these segments is considered to be 5 as sensory function in the corresponding dermatomes is intact. Of note, because weakness in the right elbow flexors/extensors is much more rostral to the bilateral sensory levels of T6 (and left motor level of T6), it is recommended that the examiner evaluates for a non-SCI condition that may be the cause of the upper extremity motor impairment. The left motor level is T6 (following the sensory level) because all motor function is presumed to be intact through this level.

#### NLI:

The NLI is C5 because this is the most rostral of the sensory and motor levels.

#### Completeness:

There is sparing of sensory function in the lowest sacral segments as S4-5 LT sensation is preserved bilaterally, so this is an incomplete injury.

#### AIS:

The AIS grade is D. This case meets criteria for a motor incomplete lesion. Even though VAC is absent, there is sensory sacral sparing and preserved motor function more than 3 levels below the motor level on both the right and left sides. Because at least half (in this case 11/18) of the key muscles below the NLI (C5) have a motor grade ≥3, this injury is classified as AIS D.

#### Sensory ZPPs:

The sensory ZPP is NA bilaterally because there is sensory sacral sparing on both sides (LT sensation is preserved at the left and right S4-5 dermatomes).

#### Motor ZPPs:

The motor ZPP, which is applicable bilaterally in the absence of VAC, is L4 on the right and S1 on the left because these are the most caudal segments on the respective sides with preserved motor function.

### Case 2: Use of the ZPPs and classification of a motor incomplete injury with only VAC present in the lowest sacral segments.

**Figure:**
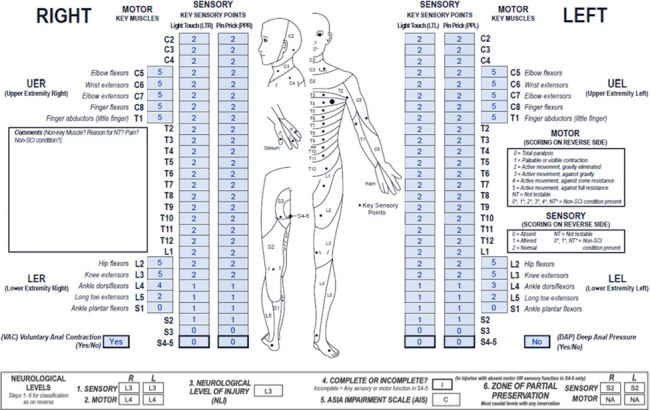
This case represents a lumbar motor incomplete SCI with sensory ZPPs that are applicable due to absent sensory sacral sparing and motor ZPPs that are NA due to preserved VAC.

#### Sensory levels:

The sensory level is L3 bilaterally as sensory function is intact from C2 through this dermatome on both sides.

#### Motor levels:

The motor level is L4 bilaterally as this is the most caudal key muscle on each side with a grade ≥3, and all motor function above this level is presumed to be intact.

#### NLI:

The NLI is L3 as this is the most rostral of the sensory and motor levels.

#### Completeness:

Although there is no sparing of sensory function in the lowest sacral segments, there is motor sacral sparing (VAC is preserved), so this is an incomplete injury.

#### AIS:

The injury severity is motor incomplete (at least AIS C) because VAC is present. This injury is classified as AIS C (and not AIS D) because less than half (in this case only 2/6) of key muscles below the NLI (L3) have a motor grade ≥3. This is an example of a rare case in which VAC is present while S4-5 PP/LT and DAP sensation are absent (reported in 1.4% of motor incomplete injuries^21^). If results of the ISNCSCI exam reveal this scenario, it would be worthwhile to carefully evaluate the rectal exam findings to ensure VAC is indeed present and that a reflex contraction of the external anal sphincter was not mistaken for a voluntary contraction.

#### Sensory ZPPs:

The sensory ZPPs are applicable because there is no sensory function in the lowest sacral segments. The sensory ZPP is S2 bilaterally as this is the most caudal segment on both sides with partially preserved sensory function.

#### Motor ZPPs:

The motor ZPP is NA bilaterally because VAC is present.

### Case 3: Use of the ZPPs and classification of a motor incomplete injury in the presence of non-key muscle function.

**Figure:**
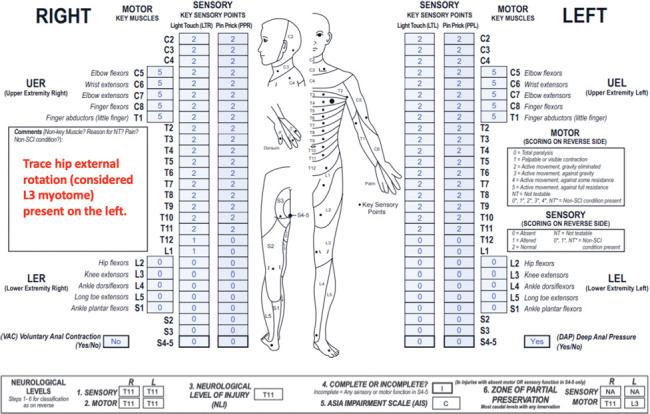
This case represents a thoracic motor incomplete SCI with sensory ZPPs that are NA due to preserved DAP sensation and motor ZPPs that are applicable because VAC is absent. An important concept in this case is recognition of nonkey muscle function and its role in the determination of AIS B versus AIS C grade and the motor ZPP.

#### Sensory levels:

The sensory level is T11 bilaterally as sensory function is intact from C2 through this dermatome on both sides.

#### Motor levels:

The right and left motor levels are T11 (following the sensory levels) because testable motor function is intact in the C5-T1 myotomes and is presumed to be intact through T11 based on intact sensory scores in all dermatomes rostral to T12.

#### NLI:

The NLI is T11 as each of the motor and sensory levels is T11.

#### Completeness:

This injury is incomplete because there is preserved DAP sensation.

#### AIS:

The AIS grade is C. Because DAP sensation is preserved, the lesion is at least sensory incomplete. VAC is absent, and there is no key muscle function more than 3 segments below the motor level on either side. However, in cases with preserved sensory function in the most caudal sacral segments, non-key muscle function should be evaluated. In this case, non-key muscle function is preserved in left hip external rotation as noted in the comments box. Hip external rotation is assigned to the L3 myotome. (Please note that the most common non-key muscle functions and their associated myotomes can be found on the back of the ISNCSCI worksheet.) Therefore, this injury is considered motor incomplete because there is sensory sacral sparing and motor sparing at left L3, which is more than 3 segments below the left motor level (T11). This injury is AIS C (and not AIS D) because less than half (in this case 0/10) of key muscles below the NLI have a motor grade ≥3.

#### Sensory ZPPs:

The sensory ZPP is NA bilaterally because there is sensory sacral sparing on both sides (DAP sensation is preserved).

#### Motor ZPPs:

The motor ZPPs are applicable because VAC is absent. On the right, the motor ZPP is T11 because there is no motor function present below the right motor level. The left motor ZPP is L3; while non-key muscle functions are not typically included in the determination process of motor ZPPs, this is a unique situation in which the presence of a non-key muscle function is the only motor finding that defines the case as motor incomplete, so the associated myotome (L3) is recorded as the left motor ZPP.

### Case 4: Use of the ZPPs and classification of a complete injury with non-SCI-related impairments rostral to the NLI.

**Figure:**
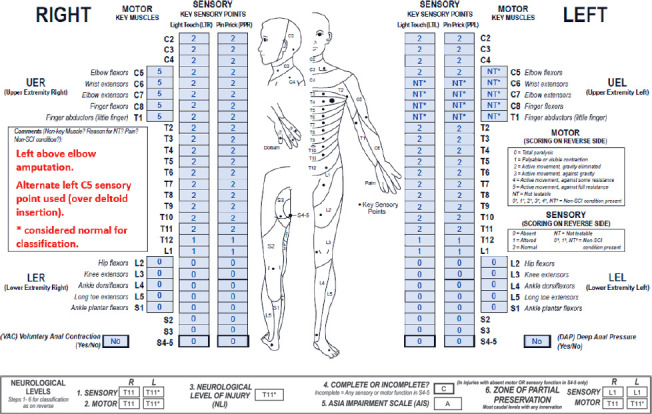
This case represents a thoracic neurological complete (AIS A) SCI in the presence of a left above-elbow amputation. As documented in the comments box, dermatomes and myotomes impacted by the amputation are graded as not testable (NT)* and are considered normal for classification because these segments are clearly located above the neurological level of injury (NLI). The NT scores require the “*”-tag as they are the result of a non-SCI condition. Sensory and motor ZPPs are applicable because this is a complete injury with no sacral sparing, and documentation of the left motor ZPP is impacted by the non-SCI condition.

#### Sensory levels:

The right sensory level is T11 because right-sided sensory function is intact from C2 through this dermatome. The left sensory level is T11*. Apart from the levels affected by the amputation, sensory function on the left is also intact from C2 through the T11 dermatome. Sensation is NT* at dermatomes C6-T1 on the left due to the amputation and therefore requires the “*”-tag. Note that because the left C5 key sensory point was also impacted by the amputation, an alternate point within the C5 dermatome was tested by the examiner as documented in the comments box. It would be best practice to include the location of the alternate testing point so that it can be used for subsequent examinations, if the non-SCI condition is permanent, as it is in this case. If an alternate sensory point is used due to a transient condition that prevents testing at the key sensory point, such as the presence of a cast, the key sensory point should be used whenever possible for follow-up exams. Based upon clinical assumptions, “*”-tagged scores are considered normal for classification, and due to this assumption, T11 requires an “*” (T11*). Under different assumptions about the dermatomes that cannot be tested, the left sensory level could be as rostral as C5.

#### Motor levels:

The right motor level defers to the sensory level of T11 because motor function is presumed to be intact through this segment. Similarly, left-sided motor function is also presumed to be intact through T11. Left C5-T1 myotomes are rated NT* due to the amputation, but the clinical assumption is that these scores are normal for classification; under different assumptions about the myotomes that cannot be tested, the motor level could be as rostral as C4. Because of this assumption, the left motor level must be tagged with an “*” (T11*).

#### NLI:

The NLI is T11*. An “*” is required for the same reasons the left sensory and motor levels require one. If the clinical assumption was that the C6-T1 dermatomes and C5-T1 myotomes would not test as intact due to the SCI (even without the amputation), the NLI could be as rostral as C4.

#### Completeness:

There is no sacral sparing, so this is a complete injury.

#### AIS:

The AIS grade is A because this is a complete injury. Note that the AIS grade is not tagged with an “*” because the presence of the non-SCI condition has no impact on the anorectal exam in this case.

#### Sensory ZPPs:

The sensory ZPP is L1 bilaterally as this is the most caudal segment on both sides with any sensory function. It is important to note that the left sensory ZPP should not receive an “*” because this classification component is not impacted by a clinical assumption; in other words, left L1 is the lowest dermatome with any sensory sparing, and this does not depend on the presence or absence of the non-SCI condition.

#### Motor ZPPs:

The motor ZPP is T11 on the right because this is the right motor level, and there is no motor function below this segment. Similarly, the left motor ZPP is T11* as this is also the left motor level, and there is no motor function distal to this myotome. The left motor ZPP requires the “*”-tag because if the assumption was instead that the C5-T1 myotomes were impaired from the SCI, the left motor ZPP could be as rostral as C4.

When determining if a classification variable requires an “*”, the International Standards Committee recommends the following steps^14^:

1. First perform the classification by replacing the “*”-tagged scores with the assumed ones.
2. Record the classification results in the respective boxes at the bottom of the ISNCSCI worksheet.
3. Next, use the actual examined scores and reclassify.
4. All differing classification variables should receive an “*”.

These steps are illustrated in the classification of Case 4:

1. LT and PP scores in the left C6-T1 dermatomes are assumed to be intact (2/2) for classification, and the motor scores in the left C5-T1 myotomes are also assumed to be intact (5/5).
2. By replacing the “*”-tagged scores with assumed ones, the right and left sensory levels are T11, the right and left motor levels are T11, the NLI is T11, this is a complete injury, the AIS grade is A, the right and left sensory ZPPs are L1, and the right and left motor ZPPs are T11. These results are recorded in the respective boxes at the bottom for the ISNCSCI worksheet.
3. Using the actual examined scores, the right sensory level is T11, the left sensory level is ND as it could be as rostral as C5, the right motor level is T11, the left motor level is ND as it could be as rostral as C4, the NLI is ND as it could be as rostral as C4, this is a complete injury, the AIS grade is A, the right and left sensory ZPPs are L1, the right motor ZPP is T11, and the left motor ZPP is ND as it could be as rostral as C4.
4. All differing classification variables, including the left sensory and motor levels, NLI, and left motor ZPP, should receive an “*”.

### Case 5: Use of the ZPPs and classification of a motor incomplete injury with non-SCI-related impairments rostral to the NLI.

**Figure:**
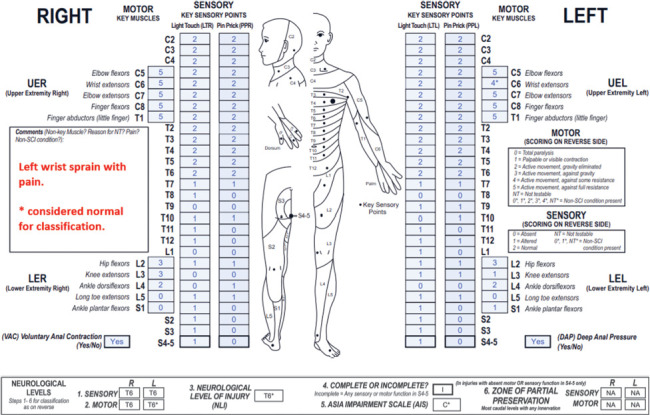
This case represents a thoracic motor incomplete SCI in the setting of a left wrist sprain and significant pain. As documented in the comments box, the myotome impacted by the non-SCI condition (left C6) is considered normal for classification. Sensory and motor ZPPs are NA due to sensory and motor sacral sparing.

#### Sensory levels:

The sensory level is T6 bilaterally as sensory function is intact from C2 through this dermatome on both sides. Note that the presence of the non-SCI condition does not affect the sensory scores or sensory levels in this case.

#### Motor levels:

Motor function is presumed to be intact from C2 through T6 bilaterally; both motor levels defer to the sensory levels of T6. Motor function is rated 4* at the left C6 myotome due to the wrist sprain and pain, and it is assumed that motor function at this level is normal for classification.

Because of this assumption, the left motor level must be tagged with an “*” (T6*). Otherwise, based on the examined scores, the left motor level would be C6.

#### NLI:

The NLI is T6*. An “*” is required for the same reason that the left motor level requires one. Based on the examined scores (without the clinical assumption), both the left motor level and NLI would be C6.

#### Completeness:

This injury is incomplete as there is sacral sparing.

#### AIS:

The AIS grade is C* because VAC is present, and less than half (3/10) of key muscles below the NLI (T6*) have a motor grade ≥3. The AIS grade requires the “*”-tag because the classification is based on the clinical assumption that the left C6 myotome would be normal if not for the non-SCI condition. Based on the examined scores alone (without the clinical assumption), the left motor level and NLI would be C6. In this scenario (NLI of C6), the AIS grade would be D because more than half (9/16) of the key muscles below the NLI would have a motor grade ≥3. The C* indicates that a clinical assumption has been made and played a role in the classification.

#### Sensory ZPPs:

The sensory ZPP is NA bilaterally because there is sensory sacral sparing on both sides (preservation of DAP and LT sensation at the S4-5 dermatomes bilaterally).

#### Motor ZPPs:

The motor ZPP is NA bilaterally because there is preserved motor function in the most caudal sacral segments (VAC is present).

### Case 6: Use of the ZPPs and classification of a complete injury with non-SCI-related impairments caudal to the NLI.

**Figure:**
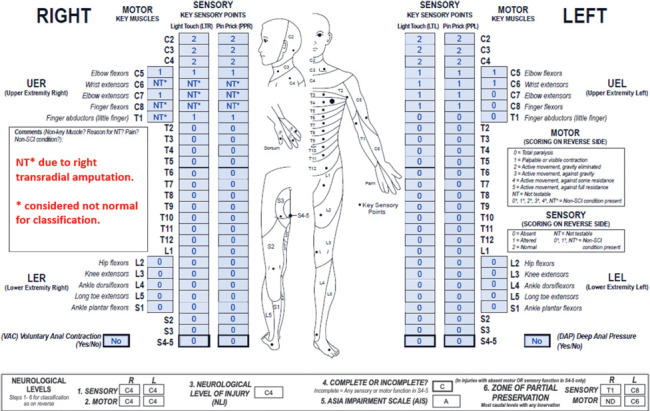
This case represents a cervical neurological complete (AIS A) SCI in the presence of a transradial amputation. As documented in the comments box, dermatomes and myotomes impacted by the amputation are graded as NT*. The “*”-tagged scores are considered not normal for classification because they are located caudal to the NLI, and it can be assumed that in the absence of the amputation, these scores would have been impacted by the SCI. Sensory and motor ZPPs are applicable because this is a complete injury with no sacral sparing, and documentation of the right motor ZPP is influenced by the non-SCI condition.

#### Sensory levels:

The sensory level is C4 bilaterally because sensory function is intact from C2 through this dermatome on each side.

#### Motor levels:

Both motor levels are also C4 because strength in the left elbow flexors (C5) is graded as only 1 bilaterally; the motor level defers to the sensory level of C4 as it is presumed that motor function is intact from C2 through this level.

#### NLI:

The NLI is C4 as each of the motor and sensory levels is also C4.

#### Completeness:

There is no sacral sparing, so this is a complete injury.

#### AIS:

The AIS grade is A because this is a complete injury. Note that the AIS grade is not tagged with an “*” because the presence of the non-SCI condition has no impact on the anorectal exam in this case.

#### Sensory ZPPs:

The sensory ZPP is applicable bilaterally in the absence of sensory sacral sparing. The sensory ZPP is T1 on the right and C8 on the left as these are the lowest levels on the respective sides with preserved sensory function. It is important to note that the right sensory ZPP should not receive an “*” because this classification component is not impacted by a clinical assumption; in other words, right T1 is the lowest dermatome with any sensory sparing, and this does not depend on the presence or absence of the non-SCI condition.

#### Motor ZPPs:

The right motor ZPP is ND due to the non-SCI condition. Based on the examination, C7 is the most caudal myotome with any motor function. However, the C8 and T1 myotomes are graded as NT*. In the absence of the amputation, it is possible that motor function would have extended to the T1 segment. Because the most caudal extent of motor preservation remains unknown, ND must be recorded for the right motor ZPP; ND does not require an “*” as this designation is not based on a clinical assumption. The left motor ZPP is C6 as this is the lowest myotome with any motor function on that side.

### Case 7: Use of the ZPPs and classification of a motor incomplete injury with non-SCI-related impairments caudal to the NLI.

**Figure:**
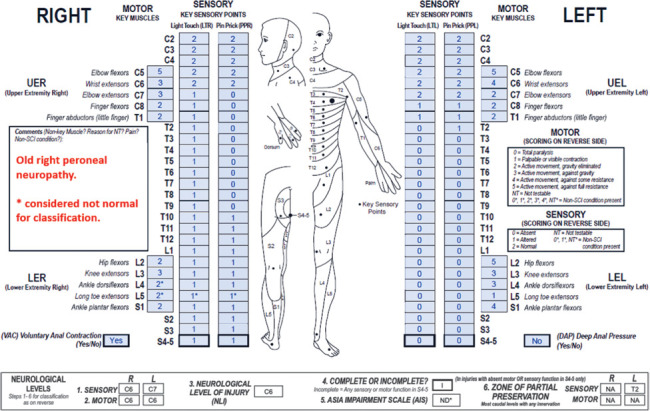
This case represents a cervical motor incomplete SCI in the presence of an old right peroneal nerve injury. As documented in the comments box, sensory and motor scores impacted by the non-SCI condition (right L5 dermatome and L4-5 key muscles) are considered not normal for classification as it is likely they are also impaired due to the SCI. The right sensory and bilateral motor ZPPs are NA due to preserved right S4-5 LT/PP sensation and intact VAC, whereas the left sensory ZPP is applicable because there is no sensory sacral sparing on that side.

#### Sensory levels:

The sensory level is C6 on the right and C7 on the left as sensory function is intact from C2 through these dermatomes on the respective sides.

#### Motor levels:

The motor level is C6 bilaterally as this is the most caudal key muscle on both sides with a motor grade ≥3, and motor function above this segment is presumed to be intact.

#### NLI:

The NLI is C6 as this is the most rostral of the sensory and motor levels.

#### Completeness:

This injury is incomplete as there is sacral sparing.

#### AIS:

While this injury is motor incomplete, the AIS grade cannot be defined and should be recorded as ND*. Based on the examined scores, less than half (6/16) of the key muscles below the NLI (C6) have a muscle grade ≥3 (which would result in an AIS grade of C). However, two of these motor scores (right L4 and L5) are graded 2* due to the presence of an old peroneal neuropathy. Although it is assumed that these scores are not normal for classification, the motor scores could be graded as 3 or 4 in the absence of the non-SCI condition. If that were the case, the AIS grade would be D as half (8/16) of the key muscles below the NLI would have a grade ≥3. Because the AIS grade could be either a C or D in the absence of the non-SCI condition, ND* must be documented. Note that the ND requires an “*” because there is an assumption that the clinical impairment is caused by both the SCI and the peroneal neuropathy; if the impairment had been caused by the SCI alone, the AIS grade would be C. Please note that it is good practice to document in the comments box that the injury is motor incomplete and that the AIS grade could be C or D.

#### Sensory ZPPs:

The right sensory ZPP is NA because there is sparing of LT and PP sensation at the right S4-5 dermatome. The left sensory ZPP is applicable in the absence of both DAP and left S4-5 LT/PP sensation, and it is T2 as this is the most caudal segment on that side with any sensory function.

#### Motor ZPPs:

The motor ZPP is NA bilaterally because there is preserved motor function in the most caudal sacral segments (VAC is present).

## Discussion

The ISNCSCI will continue to evolve as our understanding of SCI increases and advances are made in the field. The International Standards Committee considers feedback from the SCI community and implements revisions judiciously with the goals of maintaining consistency between ISNCSCI editions, improving accuracy, and increasing the classification system's utility and its inclusiveness in respect to classifiable conditions.

The 2019 ISNCSCI revision^2^ introduced these two key concepts, including broader applicability of the ZPPs and new taxonomy for non-SCI-related impairments. The sensory and motor ZPPs, which were previously only applicable in complete injuries, can now also be defined independently in incomplete injuries with absent sensory and motor sacral sparing, respectively. This change has increased the clinical value, such as the prognostic capability, of the ZPPs.^15^,^16^ The revised taxonomy for non-SCI conditions has several advantages as well. For example, the current system now includes a method for documenting sensory impairments that are presumably due to a non-SCI condition, whereas the previous system (using 5*) was limited to the motor examination. Additionally, by recording actual examined scores (as opposed to 5*), valuable clinical information is preserved, and comparisons can be made with serial assessments. Articles describing the revised definitions of the ZPPs^16^ and non-SCI taxonomy^14^ have been published by the International Standards Committee to supplement the ISNCSCI booklet and provide a more in-depth discussion of the rules and rationale for the 2019 changes.

As observed with previous ISNCSCI revisions, we expect the 2019 updates to be slowly adopted into practice. These newer concepts may not be self-explanatory and therefore require training and practice. This article and the complete ISNCSCI workbook^17^ can help to reinforce classification rules and challenging concepts. Furthermore, additional publications, webinars, and conference contributions are needed to establish these meaningful changes within the field of SCI medicine.

## Conclusion

The ISNCSCI is a continually evolving examination and classification system that is used to characterize sensorimotor deficits after SCI. The seven cases presented in this article feature key concepts from the 2019 revision. Specifically, the applicability of sensory and motor ZPPs in both complete and incomplete injuries and a new taxonomy for the documentation of non-SCI conditions are reviewed. These cases and the full ISNCSCI workbook,^17^ along with completion of the International Standards Training E Program (InSTeP),^22^ can serve as valuable training tools to improve classification accuracy. The International Standards Committee recognizes the importance of continued education and open access resources to enhance the consistency for which the 2019 changes are incorporated into everyday practice and to ensure successful utilization of this refined classification system.
